# Toxic epidermal necrolysis in hepatitis A infection with acute-on-chronic liver failure: Case report and literature review

**DOI:** 10.3389/fmed.2022.964062

**Published:** 2022-09-23

**Authors:** Xin Zang, Si Chen, Lin Zhang, Yongzhen Zhai

**Affiliations:** ^1^Department of Infectious Disease, Shengjing Hospital of China Medical University, Shenyang, China; ^2^Department of Neurology, The Fourth Affiliated Hospital of China Medical University, Shenyang, China

**Keywords:** toxic epidermal necrolysis, acute-on-chronic liver failure, hepatitis A virus, liver cirrhosis, case report

## Abstract

Toxic epidermal necrolysis (TEN) and Stevens–Johnson syndrome (SJS) are acute inflammatory skin adverse reactions characterized by epidermal exfoliation and multi-site mucositis and are considered medical emergencies. The risk factors for SJS/TEN include immune disorders, malignancy, and genetic susceptibility. In most cases, medication is considered to be the leading cause of TEN. In addition, several studies suggest that infections, such as the herpes simplex virus, human immunodeficiency virus (HIV), *Mycoplasma pneumoniae*, streptococcus, and meningococcus infections, can trigger the occurrence of SJS/TEN. In this rare case, we share our experience managing TEN in a hepatitis A virus infection with an acute-on-chronic liver failure patient. A 38-year-old man was infected with hepatitis A virus on the basis of liver cirrhosis and progressed to acute-on-chronic liver failure. As the infection progressed, the target-like skin lesions accompanied by mucosal involvement worsened. The condition of the patient progressively worsened with a severe generalized rash, bullae, and epidermal detachment accompanied by severe erosive mucosal lesions. His skin detachment area gradually involved 30% of the body surface area (BSA), and the disease progressed to TEN. The intravenous infusion of corticosteroids alleviated the patient's hypersensitivity, and the patient obtained lasting remission without severe adverse reactions and complications.

## Introduction

Stevens–Johnson syndrome (SJS) and toxic epidermal necrolysis (TEN) are acute, severe skin adverse reactions characterized by epidermal loss and multisite mucositis, accompanied by systemic disturbance (1). Both have the same risk factors and causes, which can be distinguished based on the proportion of the affected body surface area (BSA). Among them, patients with an epidermal detachment of <10% of BSA are diagnosed with SJS. Detachment of the epidermis of 10–30% of BSA is diagnosed with the overlapping group of SJS/TEN, whereas the involvement of more than 30% of BSA is diagnosed with TEN. It is reported that the incidence of SJS/TEN is about 1–3 cases per million per year (2). Although rare, the average mortality rate for SJS is <10%, and the mortality rate for TEN is more than 30%. The mortality rate is even higher in patients with sepsis, elderly patients, and patients with a sizeable epidermal detachment area at admission (3). In most cases, medication is considered to be the leading cause of TEN (4). In addition, several studies have proven that viruses, mycoplasma, and bacterial infections are all triggers of SJS/TEN, and the etiology of a few cases is still unclear.

This study reports a case of TEN caused by hepatitis A virus infection with acute-on-chronic liver failure. To our knowledge, this is the first case report to discuss TEN in acute-on-chronic liver failure secondary to acute hepatitis A infection.

## Case presentation

**Figure 1:**
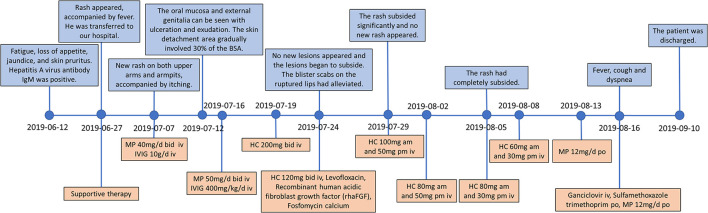
Timeline of the clinical events and treatment strategies. MP, methylprednisolone; HC, hydrocortisone; IVIG, intravenous immunoglobulin.

In June 2019, a 38-year-old male patient presented to a local hospital due to fatigue, loss of appetite, jaundice, skin pruritus, and a recent weight loss of about 7 kg. The patient denied a history of chronic diseases and relevant infectious diseases. The abdominal CT showed liver cirrhosis, splenomegaly, and varicose veins. The laboratory tests found that the hepatitis A virus antibody IgM was positive, and the total bilirubin was 392.5 μmol/L. The alanine aminotransferase was 390 U/L, and the aspartate aminotransferase was 198 U/L. He was given hepatoprotective therapy with a coenzyme complex. The coenzyme complex was a compound preparation that mainly contained coenzyme A, coenzyme I [nicotinamide adenine dinucleotide (NAD)], and reduced glutathione (GSH), but the patient's liver function did not significantly alleviate. Hematochezia occurred after 3 days, and the bleeding was reduced after hemostatic treatment with aminocaproic acid and octreotide. After 15 days, rashes appeared on the whole body, accompanied by fever with a maximum temperature of 39.0°C, and he was then transferred to our hospital for further treatment on 27 June. The timeline of the clinical events and treatment strategies is shown in Figure 1.

**Figure 2:**
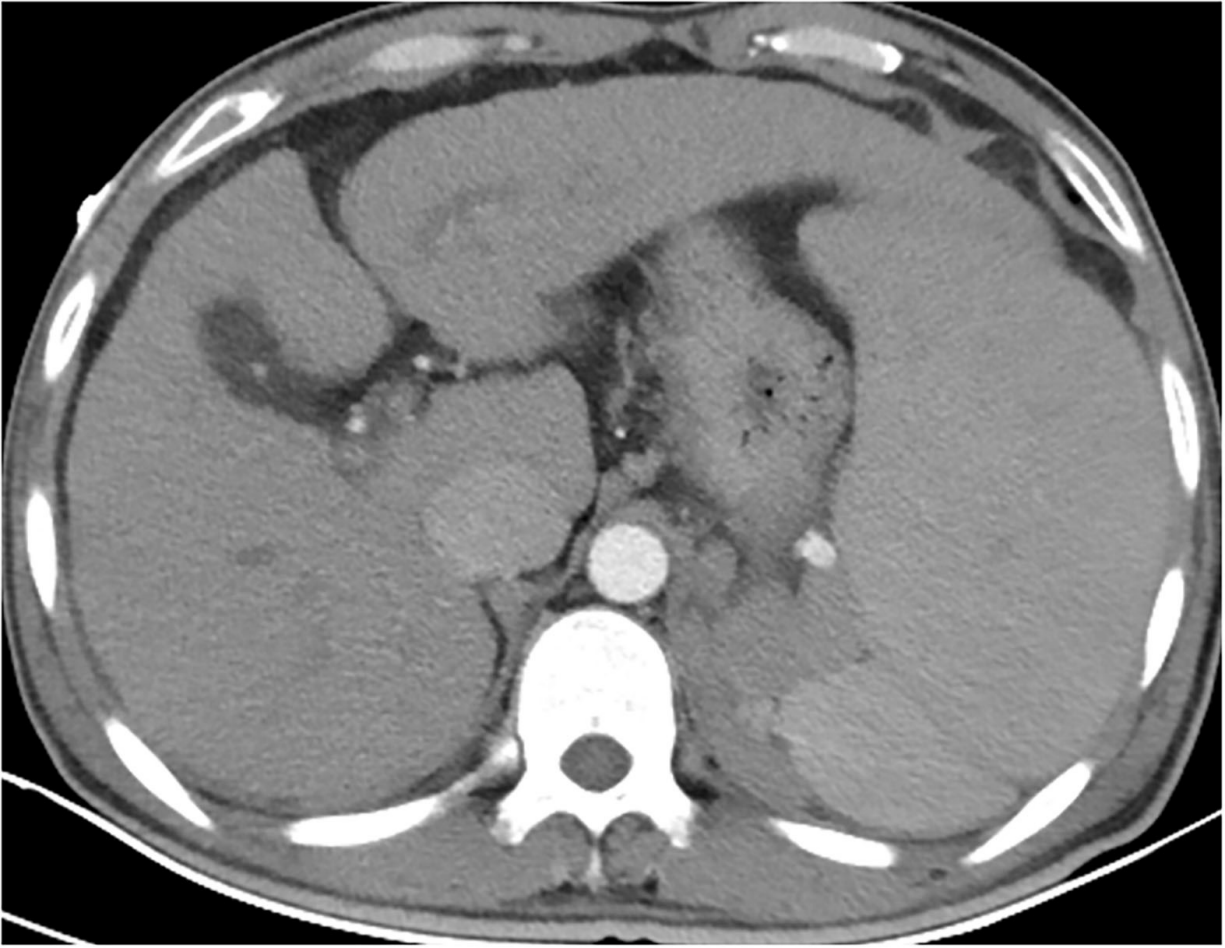
Contrast-enhanced CT of the whole abdomen revealed liver cirrhosis, splenomegaly, ascites, and portal hypertension. Varicose veins in the lower part of the esophagus. Varicose veins around the fundus of the stomach and around the spleen (2 July).

At the time of admission, the patient was lethargic, with a heart rate of 90 beats/min, a respiratory rate of 18 breaths/min, and hypotension of 97/56 mmHg. In addition, he had severe jaundice, icteric sclera, and liver palms. There were densely distributed congested maculopapular rashes on the trunk; however, the skin between the rashes was normal. The routine blood test results showed decreases in white blood cell count (1.31 × 10^9^/L), hemoglobin count (99 g/L), red blood cell count (3.04 × 10^12^/L), and platelet count (61 × 10^9^/L). As for blood biochemical parameters, the laboratory found an increase in alanine aminotransferase (65 U/L), aspartate aminotransferase (84 U/L), total bilirubin (372.8 μmol/L), conjugated bilirubin (281.2 umol/L), prothrombin time (17.50 s), and international-normalized-ratio (INR, 1.6), as well as a decrease in total protein (45.3 g/L), and albumin (24.6 g/L). He was diagnosed with decompensated liver cirrhosis, acute hepatitis A, and lower gastrointestinal bleeding (Figure 2). As the liver function worsened, according to the consensus recommendations of the Asian Pacific Association for the Study of the Liver (APASL) (5), the disease progressed to acute-on-chronic liver failure. Meanwhile, the patient denied ever having had a skin rash before and had no family history of the condition as well. He received supportive treatment with fluid supplementation, skin care, and regular infection screening. The skin was regularly flushed with sterile water, but the rash did not alleviate significantly.

**Figure 3:**
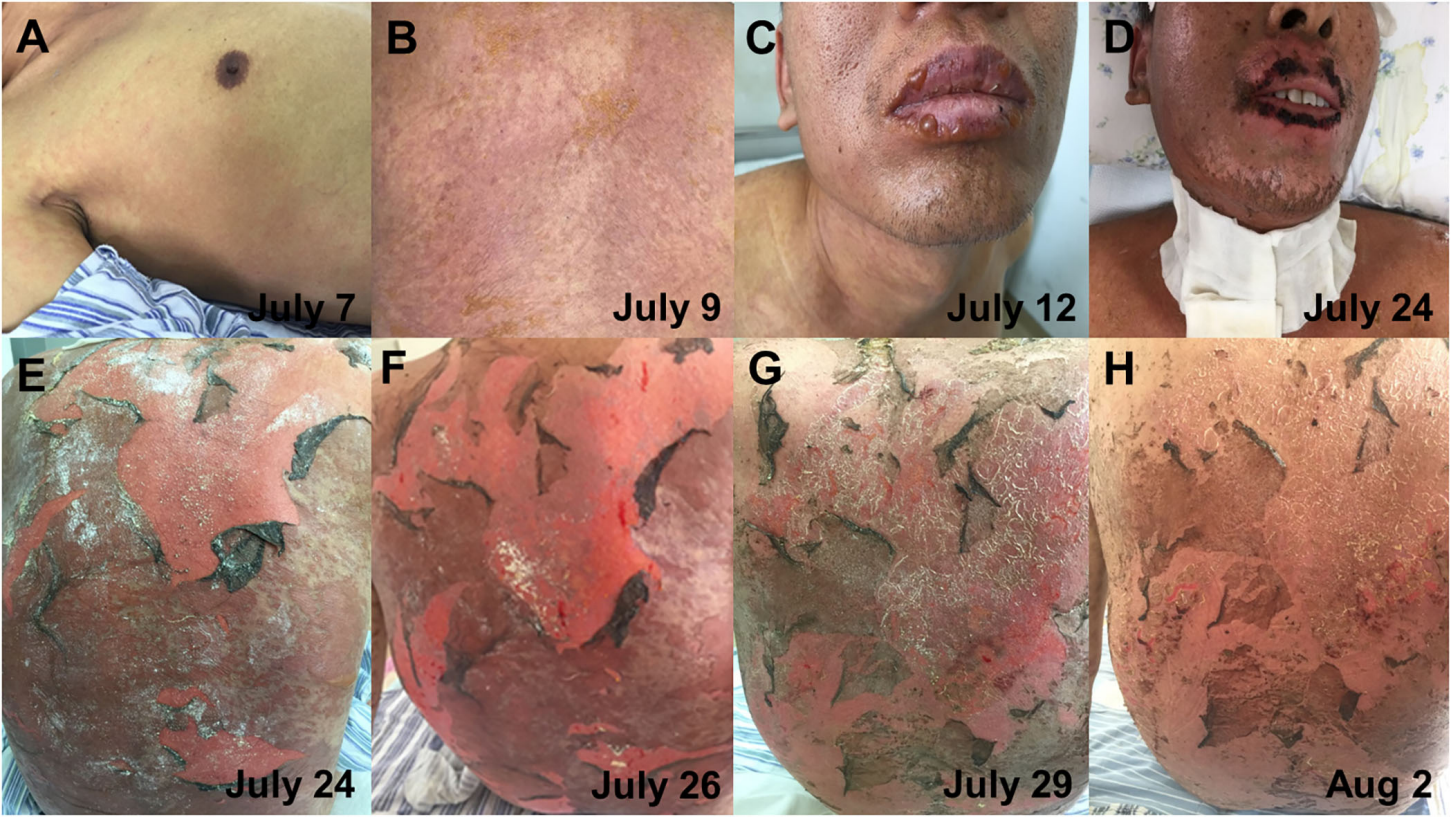
The progression of skin lesions. **(A)** The patient developed a new congestive maculopapular rash, mainly on the trunk (7 July). **(B)** The patient has a dense rash on the back (9 July). **(C)** Blisters appeared on the lips (12 July). **(D)** The scab on the lips had improved (24 July). **(E,F)** Diffuse erythema and epidermal detachment on the back (24 and 26 July). **(G,H)** The detachment of the epidermis on the back gradually improved (29 July and 2 August).

On 7 July, his symptoms showed a clinical deterioration. He developed a new congestive maculopapular rash on the inner sides of both upper arms and both sides of the armpits, with severe involvement of the palms of the hands and soles of the feet. In addition, the diffuse congestive rash was seen mainly on the trunk, accompanied by itching (Figure 3A). The patient was treated with methylprednisolone (40 mg, bid) combined with intravenous immunoglobulin (IVIG, 10 g/day) in a separate intensive care unit.

On 12 July, the patient complained of a new rash that gradually worsened, and the rash was more severe in the areas of pressure (buttocks and back), on which a positive Nikolsky sign can be induced by mechanical pressure on the skin. Blisters first appeared on the hands, feet, and lips and gradually spread to the limbs, shoulders, and neck (Figure 3C). The blisters ulcerated on their own, accompanied by pain and a burning sensation. Meanwhile, the oropharyngeal mucosa was swollen, and the patient had difficulty opening the mouth and swallowing due to repeated bleeding and crusting. Moreover, the oral mucosa and external genitalia could be seen ulcerated and exuded. The patient's condition further worsened, the skin detachment area gradually involved 30% of the BSA, and the disease further progressed to TEN. On 15 July, the patient complained of dryness in both eyes and a foreign body sensation in the blinking eyes. The ophthalmologist's examination revealed bilateral conjunctival congestion and edema, yellow sclera, and corneal transparency. The patient was diagnosed with conjunctivitis and was given levofloxacin eye drops as an anti-inflammatory treatment. In addition, daily eye care was performed to clear inflammatory secretions. On July 16, the patient received a high intravenous dose of methylprednisolone (50 mg, bid) and IVIG (400 mg/kg/day). However, the patient did not alleviate significantly. Since the therapeutic effect of intravenous methylprednisolone and IVIG was not obvious, starting from 19 July, the patient received an intravenous injection of hydrocortisone (200 mg, bid). After 5 days, there was no further necrosis and exfoliation of the skin rash and no new lesions (Figures 3D,E). Meanwhile, there was no obvious increase in external genital mucosal erosion and skin breakdown in the buttocks, and the pain was slightly reduced. Due to the massive exfoliation of the patient's epidermis, levofloxacin was added to prevent skin infections. Lyophilized Recombinant Human Acidic Fibroblast Growth Factor (rhaFGF) was used with infrared physiotherapy to promote wound healing, and fosfomycin calcium was used to prevent wound infection. Considering that the disease was under control, hydrocortisone has been applied in sufficient quantities for 5 days, and the dosage was reduced with the aim of transitioning to oral administration and eventually discontinuation. On 31 July, the rash on the face and neck completely subsided. The blisters and erosions of the external genital mucosa were obviously improved. The ophthalmologist's examination showed mild scleral yellowing and pale conjunctiva without congestion or edema. The conjunctivitis was improved and no ocular sequelae appeared. On 5 August, the patient's rash completely subsided. On 13 August, the intravenous infusion of hydrocortisone was stopped and transitioned to oral methylprednisolone tablets. The progression of the patient's skin lesions was shown in Figure 3.

**Figure 4:**
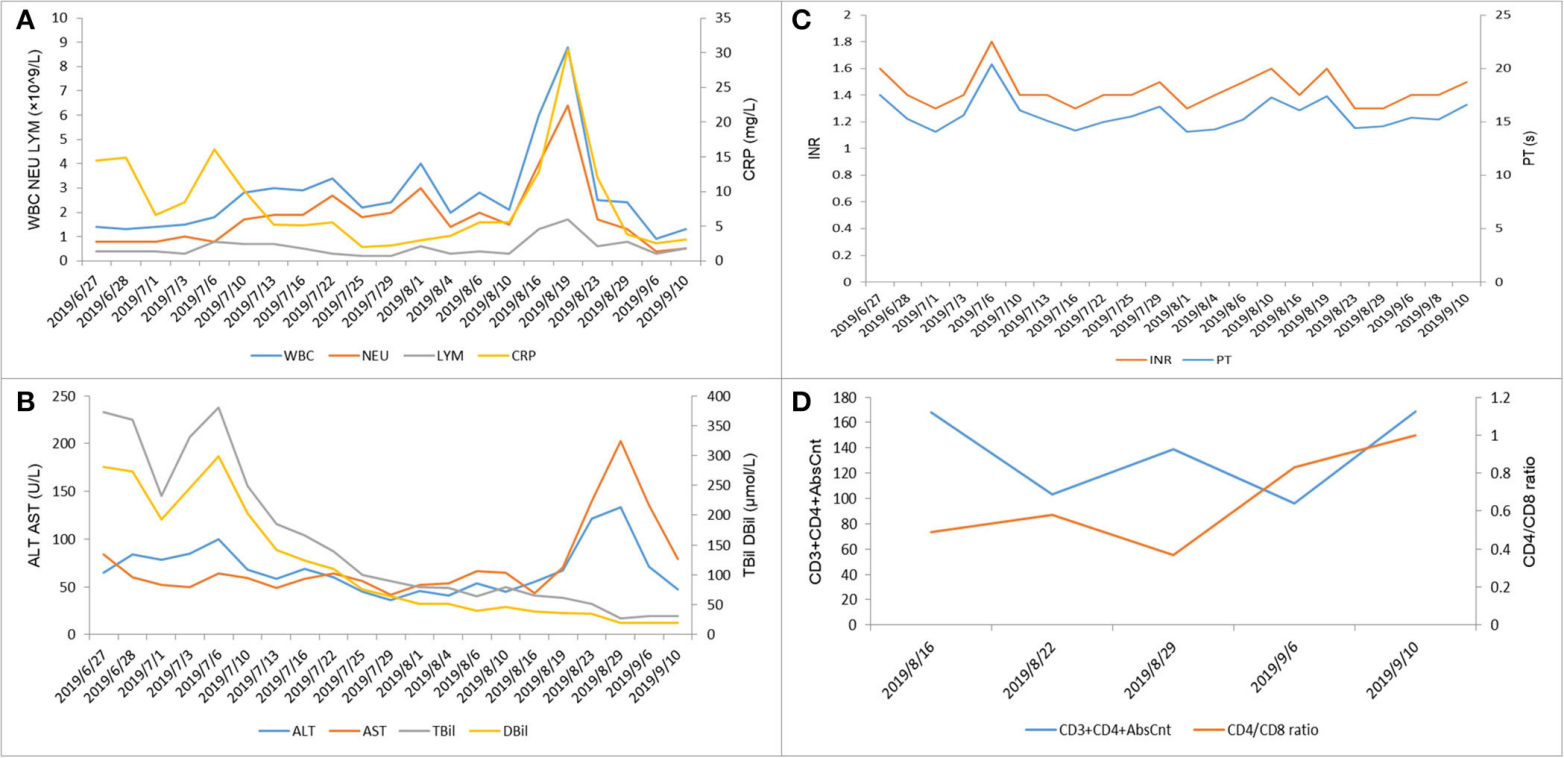
Laboratory test indicators during the hospitalization of this patient. **(A)** White blood cells (×10^9^/L), neutrophils (×10^9^/L), lymphocytes (×10^9^/L), and C-reactive protein (mg/L). **(B)** Alanine aminotransferase (U/L), aspartate aminotransferase (U/L), total bilirubin (μmol/L), and direct bilirubin (μmol/L). **(C)** International normalized ratio (INR) and prothrombin time (s). **(D)** CD3+CD4+AbsCnt and CD4/CD8 ratio.

**Figure 5:**
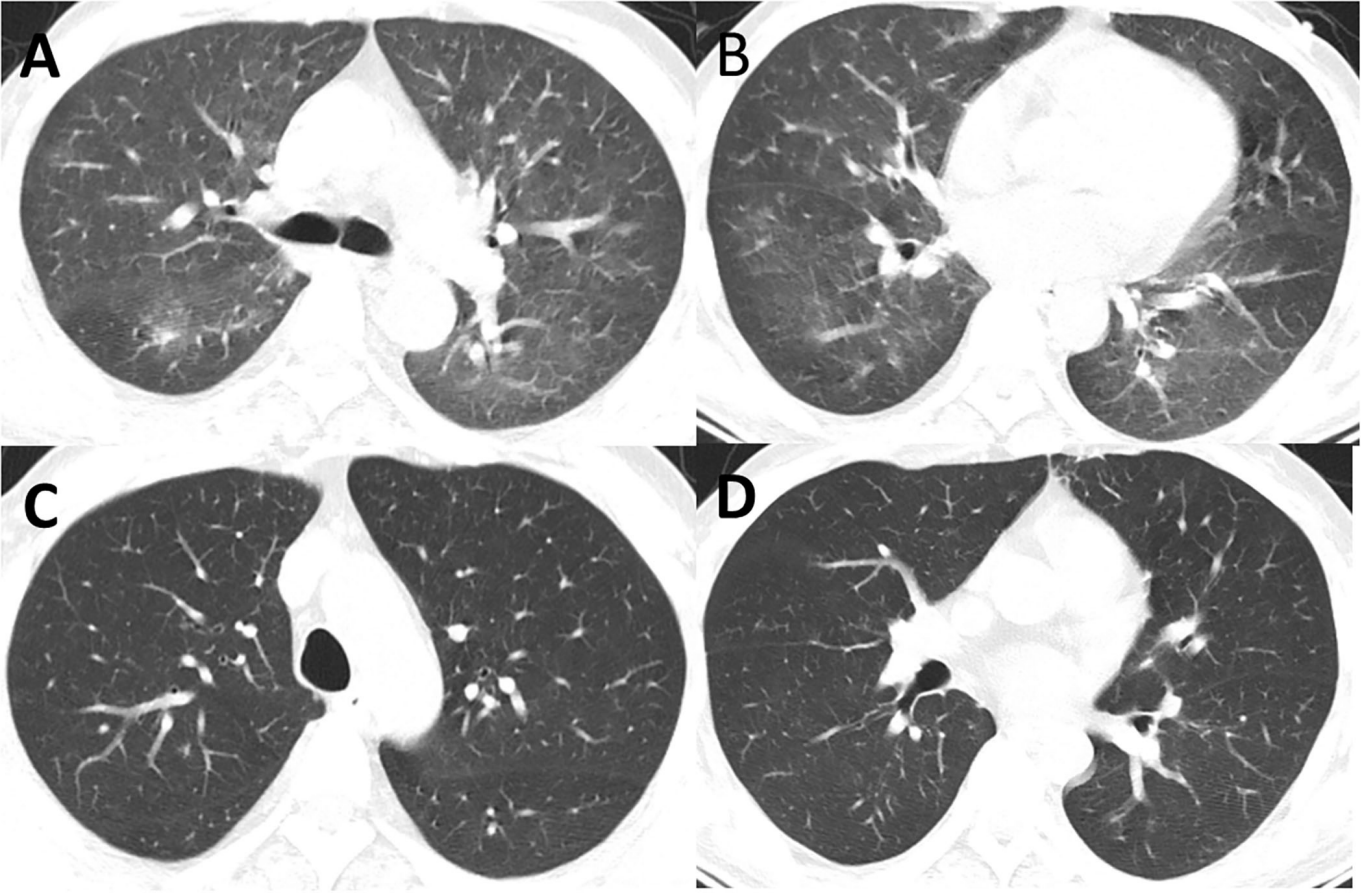
**(A,B)** CT showed new inflammation and inflammatory nodules in the lingual segment of the right lung and the upper lobe of the left lung (16 August). **(C,D)** CT showed that the double pneumonia was less than before (5 September).

On 16 August, the patient had a sudden fever, cough, and dyspnea. The body temperature rose to 37.6°C. The white blood cell count was 6.9 × 10^9^/L; the percentage of neutrophils was 65.8%, C-reactive protein was 19.4 mg/L, and the absolute count of CD4+ T cells was <200 (Figure 4D). The antibodies for Cytomegalovirus, Epstein-Barr virus, Mycoplasma pneumoniae, and Chlamydia pneumoniae were all negative. The chest CT showed ground glass-like and grid-like changes (Figure 5). Considering that the patient's weakened immune system may cause opportunistic infections, the possibility of Pneumocystis carinii infection was high. He was given an oral antibiotic sulfamethoxazole-trimethoprim, ganciclovir intravenously, combined with oral methylprednisolone tablets 12 mg/day. The cough and fever improved after the medications, and the patient's inflammation indicators gradually decreased. Furthermore, the chest CT confirmed the improved pulmonary infection. We instructed the patient to reduce methylprednisolone tablets to 2 mg/day every week until discontinuation. Important indicators were regularly reviewed throughout the treatment process (Figure 4).

After the treatment mentioned above, the patient's rash completely subsided and the function indexes of multiple organs tended to stabilize and improve before being discharged from the hospital on 10 September. After 2 months, the patient was admitted for follow-up on 5 November. As for the blood biochemical parameters, the laboratory found an increase in alanine aminotransferase (91 U/L), aspartate aminotransferase (151 U/L), total bilirubin (97.1 umol/L), conjugated bilirubin (79.3 umol/L), as well as a decrease in albumin (24.4 g/L). Meanwhile, his skin lesions had completely subsided, with no recurrence or complications and no ocular sequelae appeared.

## Discussion and literature review

Medication is considered to be the leading cause of SJS/TEN. A number of studies have also proven that infections, such as herpes simplex virus and human immunodeficiency virus (HIV), are all triggering factors for SJS/TEN (4). Since the first case of TEN caused by hepatitis A virus was reported in Israel in 1989 (6), there have been no other studies demonstrating the association between hepatitis A virus and subsequent TEN. Here, we report a case of TEN in acute-on-chronic liver failure secondary to hepatitis A. In this study, the patient was treated with hepatoprotective drugs and hemostatic medications before the rash onset, none of which were reported as suspicious high-risk pathogenic drugs in the literature. In addition, we assessed causality between these drug exposures and TEN using the Algorithm of Drug Causality in Epidermal Necrolysis (ALDEN) score (7). Based on the ALDEN score, the coenzyme complex with an ALDEN score of 1 point was classified as an unlikely cause. With an ALDEN score of −1 point, aminocaproic acid and octreotide were classified as very unlikely causes. Meanwhile, the laboratory tests have demonstrated that the patient was not infected with other pathogenic bacteria or viruses. Therefore, we believe that the patient's TEN was presumably caused by the hepatitis A virus. We will discuss our findings in terms of the following three sections, namely the pathogenesis, treatment, and infection monitoring of SJS/TEN.

### Pathogenesis of SJS/TEN

The pathogenesis of SJS/TEN is not yet fully understood, but T cell-mediated drug-specific cytotoxicity, genetic linkage, granulysin, and other mechanisms are considered to play a crucial role in the pathogenesis of SJS/TEN. The histopathological study of SJS/TEN skin lesions showed that the diffuse apoptosis of keratinocytes caused the patient's extensive epidermal detachment (8). Studies have analyzed the live immune cells in the vesicular fluid of patients with TEN and found that T lymphocytes are the primary cell type. In the early stage of the disease, cytotoxic T lymphocytes (CTL) are the most critical cells in the blister fluid (9, 10). Therefore, the occurrence of TEN may be a T cell-mediated cytotoxic reaction against keratinocytes, which leads to the apoptosis of keratinocytes. Medication is considered to be the leading cause of TEN. The drug antigen activates T cells through the interaction of T cell receptor (TCR) and MHC on antigen presenting cells, leading to the clonal expansion of CD8+ CTL. Cytotoxic T cells can induce extensive apoptosis of TEN epidermal cells through the perforin/granzyme B pathway (11). In addition, the granulysin secreted by drug-specific CTL cells and NK cells is also a key mediator of keratinocyte apoptosis in SJS/TEN (12).

Several previous studies have identified infections associated with SJS/TEN, such as the herpes simplex virus, human immunodeficiency virus (HIV), and *Mycoplasma pneumoniae*, which can trigger the occurrence of SJS/TEN. For example, the occurrence of SJS/TEN in patients with HIV is thought to be the result of multiple synergistic factors. The incidence of skin adverse reactions in patients with HIV was inversely correlated with the peripheral blood CD4+ T cell count and CD4+/CD8+ ratio in a study cohort (13). Another study suggested that HIV infection predisposed skin to TEN *via* depletion of skin-directed CD4+ T cells. Furthermore, an imbalance in the CD4+/CD8+ cell ratio may predispose the development of TEN (14). In our case, the patient was infected with hepatitis. A virus on the basis of liver cirrhosis. With the further deterioration of liver function, he developed acute-on-chronic liver failure. Patients with acute-on-chronic liver failure often show an attenuated activity of immune cells (15). In addition, our patient showed a decrease in peripheral blood CD4+ T cell count and a CD4+/CD8+ cell ratio. On the basis of current evidence, it is undeniable that infections play an important role in the formation of SJS/TEN (4). However, the pathogenesis and pathogenic mechanisms of infection leading to SJS/TEN still need further research.

Patients with SJS/TEN usually have extensive and varying degrees of mucosal involvement, especially in the eyes. Studies have shown that more than half of patients with SJS/TEN are accompanied by severe ocular complications (SOCs) (16). The acute phase is often accompanied by a variety of manifestations, such as eyelid edema, conjunctival edema or congestion, conjunctivitis, keratitis, or corneal ulceration. In the chronic stage, it manifests as severe sequelae, such as dry eye, trichiasis, and vision loss (17). It is worth noting that the pathogenesis and genetic background appear to be different in SJS/TEN patients with and without SOCs. For example, among patients with drug-related SJS/TEN, patients with SJS/TEN caused by the use of acetaminophen for the common cold showed a higher incidence of SOCs than those patients with SJS/TEN caused by the use of other drugs (18). In addition, there were racial differences in HLA types associated with SJS/TEN and SOCs. For example, HLA-A^*^02:06 is associated with Japanese and Korean patients, while HLA-B^*^44:03 is associated with Japanese, Indian, and European ancestry Brazilian patients (4, 17, 19).

In addition to cold medications, infections, such as viruses or mycoplasma, may play a triggering role in the onset of SJS/TEN with SOCs (20). In this case of toxic epidermal necrolysis caused by hepatitis A virus, the patient was diagnosed with conjunctivitis. Conjunctivitis has improved and the ocular signs and symptoms have completely subsided after the above treatment without serious ocular sequelae. Cold medicines are considered to be one of the most common factors causing SJS/TEN with SOCs. A retrospective study from Japan showed that the patient's age and the cold medicine used as the exposure drug were predictors of increased ocular severity in the acute phase. Meanwhile, the incidence of severe chronic ocular sequelae increased with the severity of the eye in the acute phase (21). In our case, however, the patient presented with relatively mild ocular symptoms and no serious sequelae developed, which could be related to hepatitis A virus infection. There are fewer studies on viral infections leading to SJS/TEN with SOCs at present, and further studies on the severity of SJS/TENS with SOCs and ocular management are needed to reduce the occurrence of serious ocular sequelae.

### Treatment of SJS/TEN

At present, there is no specific treatment for SJS/TEN that has shown effectiveness in clinical trials. Previous studies have shown that several common treatments include supportive therapy, immunosuppressive drug therapy, intravenous immunoglobulin, and plasma exchange (22). Among them, the use of methylprednisolone pulse therapy and intravenous immunoglobulin combined with methylprednisolone pulse therapy is increasing for severe and rapidly progressive cases. The effectiveness of these two treatments is still controversial. A study from India demonstrated that low-dose immunoglobulin combined with steroid therapy showed benefits in reducing mortality and preventing disease progression (23). However, another retrospective study involving 281 patients found that compared with supportive treatment, intravenous immunoglobulin and steroids had no significant effect on patient mortality (24).

Studies have shown that if high-dose glucocorticoids are given to patients with liver failure, it may increase the incidence of adverse events (25). In our case, the patient was diagnosed with acute-on-chronic liver failure after admission, and the rash was not yet typical. Therefore, the patient was given supportive treatment based on the principle of safety. Unfortunately, the rash worsened on the 10th day of admission, and the patient received an intravenous infusion of methylprednisolone (40 mg, bid) in combination with IVIG (10 g/day). Although there is no conclusive evidence that any interventions are more effective than conservative treatments, a recent case report found that as the patient's disease worsened, a gradual escalation of treatment protocol helped the patient recover from the disease (26). When the treatment is effective, the patient's skin lesions and erythema usually begin to recover within 3 days after starting the treatment (27). However, in our case, the therapeutic effect of this treatment was not obvious. In a retrospective study, the authors found that the steroid pulse therapy of two patients who died was started more than 7 days after the onset of the disease. It is speculated that the delay of treatment may weaken the effect of steroid pulse therapy (27). In another case of TEN caused by the use of amphetamine, the author believed that the delayed use of intravenous immunoglobulin may have caused the delay of the patient's re-epithelialization (28). These results suggest that early interventions may be effective and contribute to the improved prognosis of SJS/TEN. However, steroid pulse therapy does remain controversial at present, and large-scale randomized clinical trials are needed to find out the effectiveness, exact timing, and optimal dose of steroid therapy for SJS/TEN.

Liver injury is one of the common complications of SJS/TEN. Studies have shown that liver disease history is a risk factor for poor prognosis of SJS/TEN patients with liver injury (29). In our case, with the further deterioration of liver function and rash, the patient's condition worsened and the treatment became more difficult. Even worse, the patient's immune system was weak, which increased the possibility of causing sepsis. Considering that known inflammation and pro-apoptotic mechanisms play a role in SJS/TEN, there is no consensus on the selection of steroid hormones and the optimal dosage. We hypothesize that the role of steroid hormones in the clinical treatment of patients with sepsis gives us some thoughts. Studies have shown that high-dose or long-term use of methylprednisolone is not beneficial in the treatment of sepsis and may increase the incidence of adverse events (30). However, the use of low-dose hydrocortisone (<400 mg/day) will shorten the reversal time of shock, and the incidence of complications caused by steroids is relatively low (31, 32). This may be because methylprednisolone has a stronger immunosuppressive effect, while hydrocortisone has more mineralocorticoid effects and vasoactive properties. As the effect of intravenous infusion of methylprednisolone combined with immunoglobulin therapy was not obvious, after fully considering the patient's condition, the treatment was changed to intravenous hydrocortisone infusion (200 mg, bid). With the gradual improvement of the rash, the dosage of hydrocortisone was reduced to the oral dose and finally withdrawn completely.

In addition, previous case reports described the effectiveness of intravenous hydrocortisone in the treatment of SJS/TEN. In a case report from the United States, a small dose of hydrocortisone combined with vitamin C and thiamine (HAT therapy) was used to treat a patient with SJS/TEN overlap. The patient's condition was significantly improved within 48 h (33). In another case report from the United Kingdom, when the disease progressed, effectiveness was shown after the gradual escalation of the treatment protocol from oral prednisolone to intravenous hydrocortisone (26). For steroid therapy, the choice of hormones, the appropriate dosage, the ideal treatment time, and safety still need to be further explored in clinical research.

### Importance of infection monitoring

In our study, the patient developed a lung infection on 16 August, and the absolute value of CD4+ T cell count was <200. Considering that the patient's weakened immune system may cause opportunistic infections. Due to the extensive detachment of the epidermis, mucosal damage, and drug-induced immunosuppression, patients with SJS/TEN are prone to infectious complications (34), and the high mortality rate of SJS/TEN is mainly due to the occurrence of sepsis (35). In addition, studies have shown that the use of corticosteroids may increase the infection rate and mortality of patients with SJS/TEN (36). Unfortunately, our patient had acute-on-chronic liver failure. Patients with liver failure are susceptible to bacterial and fungal infections due to the immune paralysis of circulating immune cells (15). Therefore, we believe that infection monitoring is necessary for patients with SJS/TEN, especially those with underlying diseases that easily lead to low immunity or who are treated with corticosteroids. It is still controversial whether the use of corticosteroids for the treatment of SJS/TEN will bring serious negative effects on patients. Previous studies have shown that the early use of corticosteroid pulse therapy can significantly reduce the occurrence of ocular complications, and it has no significant effect on mortality and the incidence of sepsis (24, 37). Whether steroid therapy treatment of SJS/TEN will produce serious negative effects still needs further research.

Over the recent decades, we have gained a deep understanding of the immunological mechanism of SJS/TEN. With the progress in drug research, there are several reports on SJS/TEN caused by new drugs and new biological products. For instance, several cases of SJS/TEN due to COVID-19 vaccination have been reported recently, and this poses new challenges for the early diagnosis and identification of pathogenic factors of SJS/TEN (38, 39). At the same time, infection and autoimmune diseases are also potential pathogenic factors. The possible interaction between infection and drugs or the interaction between different drugs needs further study. The mortality of SJS/TEN is usually high, and its treatment is very complicated and difficult. There is currently no consensus and universally applicable treatment plan. Most studies are published in the form of case reports, systematic reviews, and meta-analyses. More large-scale randomized clinical trials are needed to formulate individualized treatment plans based on the different pathogenic factors of patients and the severity of the disease.

This study has some limitations. Unfortunately, the patient developed abnormal coagulation due to acute-on-chronic liver failure; therefore, we were unable to perform a histological examination. There is a need for further research which would provide more insight into the mechanisms and early recognition of TEN induced by the hepatitis A virus.

## Conclusion

In our study, we report a case of TEN caused by hepatitis A virus infection with acute-on-chronic liver failure. In addition, we explored the treatment of TEN in combination with acute-on-chronic liver failure for the first time, including the dose of hormones and the duration of therapy. The potential occurrence of serious cutaneous adverse events requires medical staff to be aware of such skin reactions when managing patients with the hepatitis A virus. Meanwhile, we emphasize the importance of early clinical suspicion, infection monitoring, and management by a multidisciplinary team.

## Data availability statement

The datasets for this article are not publicly available due to concerns regarding participant/patient anonymity. Requests to access the datasets should be directed to the corresponding author.

## Ethics statement

This study protocol meets the requirements of the Medical Ethics Committee of Shengjing Hospital of China Medical University. The patient provided his written informed consent to participate in this study. Written informed consent was obtained from the patient for the publication of any potentially identifiable images or data included in this article.

## Author contributions

YZ contributed to the conception and design of this study and finally approved the manuscript version to be submitted. XZ and SC analyzed the data and wrote the manuscript. LZ contributed to clinical management of the patient and constructive discussions and revised the manuscript as well. All authors contributed to the article and approved the submitted version.

## Conflict of interest

The authors declare that the research was conducted in the absence of any commercial or financial relationships that could be construed as a potential conflict of interest.

## Publisher's note

All claims expressed in this article are solely those of the authors and do not necessarily represent those of their affiliated organizations, or those of the publisher, the editors and the reviewers. Any product that may be evaluated in this article, or claim that may be made by its manufacturer, is not guaranteed or endorsed by the publisher.
